# A multiplex PCR mini-barcode assay to identify processed shark products in the global trade

**DOI:** 10.1371/journal.pone.0185368

**Published:** 2017-10-11

**Authors:** Diego Cardeñosa, Andrew Fields, Debra Abercrombie, Kevin Feldheim, Stanley K. H. Shea, Demian D. Chapman

**Affiliations:** 1 School of Marine and Atmospheric Science, Stony Brook University, Stony Brook, New York, United States of America; 2 Abercrombie & Fish, Miller Place, New York, United States of America; 3 Pritzker Laboratory for Molecular Systematics and Evolution, The Field Museum, Chicago, Illinois, United States of America; 4 BLOOM Association, Central, Hong Kong; 5 Department of Biological Sciences, Florida International University, North Miami, Florida, United States of America; University of Guelph, CANADA

## Abstract

Protecting sharks from overexploitation has become global priority after widespread population declines have occurred. Tracking catches and trade on a species-specific basis has proven challenging, in part due to difficulties in identifying processed shark products such as fins, meat, and liver oil. This has hindered efforts to implement regulations aimed at promoting sustainable use of commercially important species and protection of imperiled species. Genetic approaches to identify shark products exist but are typically based on sequencing or amplifying large DNA regions and may fail to work on heavily processed products in which DNA is degraded. Here, we describe a novel multiplex PCR mini-barcode assay based on two short fragments of the cytochrome oxidase I (COI) gene. This assay can identify to species all sharks currently listed on the Convention of International Trade of Endangered Species (CITES) and most shark species present in the international trade. It achieves species diagnosis based on a single PCR and one to two downstream DNA sequencing reactions. The assay is capable of identifying highly processed shark products including fins, cooked shark fin soup, and skin-care products containing liver oil. This is a straightforward and reliable identification method for data collection and enforcement of regulations implemented for certain species at all governance levels.

## Introduction

Monitoring the international trade of wildlife has become a priority for most countries, although enforcement is challenging due to the lack of appropriate and cost-effective implementation tools [1]. In the past few decades sharks have been heavily exploited in many fisheries around the world, mainly to supply demand for fins and meat [2–6]. The Convention on International Trade in Endangered Species of Wild Fauna and Flora (CITES) currently lists twelve shark species in Appendix II, which allows international trade of these species with proper documentation certifying that products being traded were sustainably and legally taken from the wild (*Rhincodon typus* (2001), *Cetorhinus maximus* (2001), *Carcharodon carcharias* (2004), *Carcharhinus longimanus* (2013), *Lamna nasus* (2013), *Sphyrna lewini* (2013), *S*. *mokarran* (2013), *S*. *zygaena* (2013; [7]), *Carcharhinus falciformis* (2016), *Alopias superciliosus* (2016), *A*. *pelagicus* (2016), and *A*. *vulpinus* (2016)). The adoption and enforcement of these listings by countries requires border control agents to be able to identify the species a product is made from in order to identify illicit trade (i.e., trade without permits). Beyond the enforcement of CITES regulations there are many other monitoring and law enforcement contexts where it may be necessary to identify shark species, for example when certain species are prohibited in a fishery (e.g. [8]) or when landings must be tracked by species in order to keep them below catch limits.

Several visual [9] and molecular techniques [10–14] have been developed to identify shark products at different stages along the supply chain of the trade (e.g., wet, frozen, dried unprocessed fins; dressed carcasses, meat products). Many other shark products such as liver oil, salted meat, and some processed fins cannot be visually identified to species and contain highly degraded genomic DNA that reduces amplification and sequencing success for genetic identification. To overcome this obstacle [10] developed a mini-DNA barcoding assay to identify the species of origin of heavily processed products that allows the detection of potentially illicit trade in threatened species under the control of international protections (i.e, CITES-listed shark species). This barcoding assay used a small ~ 150 base pair (bp) fragment of the cytochrome oxidase I (COI) gene to identify processed fins and fin soup to genus or species level. Despite its high success rate on processed products (79%; [10]), this mini-barcoding assay produced a short amplicon with an insufficient number of bases to effectively distinguish the CITES-listed oceanic whitetip shark (*Carcharhinus longimanus*) from the Galapagos shark (*C*. *galapagensis*), dusky shark (*C*. *obscurus*), and Caribbean reef shark (*C*. *perezi*). In addition, identification uncertainty (i.e., only to genus) was common within the commonly traded requiem sharks (*Carcharhinus spp*.) [10], which may be problematic in law enforcement contexts when these species are prohibited from fisheries and trade [15]. Here, we present a novel methodology that builds on [10] for identifying processed shark products. The new assay uses multiplex PCR to amplify up to 3 COI fragments ~150 bp, ~200 bp and, in some cases, the full COI sequence (~650 bp) simultaneously, any of which can then potentially be sequenced using one of the amplification primers. The combined 150 and 200 bp fragments, or the full COI, enables identification of the majority of shark species in trade and all of the CITES-listed species.

## Methodology

**Fig 1:**
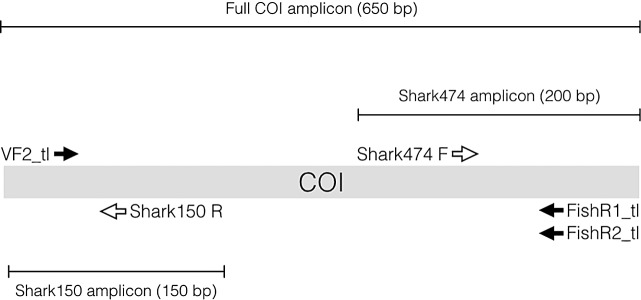
Schematic representation of the mini-barcode multiplex PCR assay with relative annealing sites and orientation of each primer, and spatial coverage of the resulting amplicons. Solid arrows denote universal primers and open arrows represent the mini-barcode primers.

We used the mini-barcode assay described in [10] as a starting point. Sharks in the Order Carcharhiniformes comprise the majority of species in the fin trade and are also frequently difficult to identify with this assay, including the CITES listed oceanic whitetip (*Carcharhinus longimanus*) and silky sharks (*C*. *falciformis*). To ameliorate this, we downloaded all of the COI sequences for the *Carcharhinus spp*. (the most speciose genus in the Order) from the National Center for Biotechnology Information (NCBI) GenBank (http://www.ncbi.nlm.nih.gov/genbank/; as of Feb 2013; S1 Table) in order to develop a second mini-barcoding primer that could be multiplexed with [10]. These sequences were aligned and a consensus sequence for each species was created in BioEdit [16]. The consensus sequences for each species were then aligned and a primer was designed in a conserved region in the 3’ end of the COI barcoding region approximately 474 bp downstream of the beginning of the sequence. We designed a novel forward primer (Shark474F 5’- CHATTTCCCAATATCAAACACC-3’) to anneal to this conserved region. When used in conjunction with two overlapping M13 tagged universal reverse primers ([17]; FishR1_t1 5’-CAGGAAACAGCTATGACACTTCAGGGTGACCGAAGAATCAGAA-3’ and FR2_t1 5’-CAGGAAACAGCTATGACACCTCAGGGTGTCCGAARAAYCARAA-3’) we anticipated that a multiplex PCR would yield an approximately 200 bp amplicon with an M13 tail from shark DNA templates. The amplicon could then be sequenced with the M13 primer and would yield a sequence of ~ 145 to 155 bp. We predicted that a multiplex of these primers and those used in [10] would potentially yield up to three amplicons: the ~150bp amplicon (from now on referred to as Shark150) from [10], the ~200bp amplicon (from now on referred to as Shark474) described above, and an amplicon of the entire COI barcoding region (~650bp; Fig 1). We initially tested and optimized a new multiplex PCR assay using one universal forward COI primer and two universal reverse COI primers and the two internal mini-barcode primers 150R and 474F. We did not include the universal forward reverse primer FishF2_t1 because initial trials indicated that it interacted with 474F and caused the latter to fail to amplify. The ability of the universal primers used in [10] to amplify the full COI from well preserved genomic DNA is well documented [17], so there was no need to test our new assay extensively with tissues of this nature. We therefore focused our testing of the assay on processed dried fin samples that generally contain more degraded DNA than preserved tissues. We quantified amplification and sequencing performance on 200 processed dried fin samples of unknown species identity from the Guangzhou dried seafood market, where we knew that a wide range of chondrichthyan species could be found based on previous testing with the Shark150 alone. The purpose of this test was to quantify how often the assay led to successful sequencing of each type of amplicon (whole COI, 150R, and 474F). These fin tissues were collected in retail markets in Guangzhou. We also tested the assay on four shark liver oil pills, ceratotrichia from four shark fin soup samples, and four samples taken from three different skin-care products containing squalene (shark liver oil), in the forms of facial foam, facial mask and facial oil, to assess performance on other types of processed products.

Genomic DNA was extracted following the protocols used by [18,19]. Briefly, a small piece of tissue (processed fin or facial mask) of approximately 2 mm^2^ was cut and placed in a PCR tube with 200 μl of 10% Chelex Resin (BioRad). Processed fin samples were agitated under water before extraction to reduce potential contamination. For the liquid samples (i.e. facial foam, facial squalene, and liver oil pills), 2 μl were used in each PCR tube with 10% Chelex. Once in Chelex, samples were heated at 60°C for 20 min and then at 99°C for 25 min, followed by a brief centrifugation. Each 25 μl PCR included 0.5 μl of extracted DNA, 12.5 μl of GoTaq® Hot Start Green Master Mix (Promega) and the five primers (volumes listed in Table 1). The multiplex PCR was amplified with the following conditions: an initial denaturation at 94°C for 2 min, followed by 35 cycles at 94°C for 1 min, 52°C for 1 min, and 72°C for 1 min, with a final extension of 72°C for 10 min. Multiplex PCRs were checked on a 3% agarose gel and all products were cleaned using ExoSAP-IT (Affymetrix, Inc., Santa Clara, CA, USA).

**Table 1:** Primer sequences and volumes use in the multiplex PCR assay. All primers were used with a concentration of 10 μM.

Primer name	Primer sequence	Volume [μl]	References
VF2_tl	5’-TGTAAAACGACGGCCAGTCAACCAACCACAAAGACATTGGCAC-3’	1.5	Ward et al. (2005)
FishR1_tl	5’-CAGGAAACAGCTATGACACTTCAGGGTGACCGAAGAATCAGAA-3’	0.75	Ward et al. (2005)
FishR2_tl	5’-CAGGAAACAGCTATGACACCTCAGGGTGTCCGAARAAYCARAA-3’	0.75	Ward et al. (2005)
Shark150R	5’-AAGATTACAAAAGCGTGGGC-3’	0.375	Fields et al. (2015)
Shark474F	5’-CHATTTCCCAATATCAAACACC-3’	0.1875	This study

All products were sequenced twice using the Big Dye Terminator v3.1 cycle sequencing kit (Applied Biosystems, Foster City, CA, USA). Sequencing was performed on an ABI 3730 DNA Analyzer (Applied Biosystems) using the M13 forward primer and the M13 reverse primer. If the whole COI barcoding region had amplified then it was sequenced with both the M13 forward and M13 reverse primer, yielding a long sequence of > 500 bp. When present, the 150R amplicon was sequenced with the M13 F primer and the 474F amplicon was sequenced with the M13R primer on separate sequencing reactions to avoid a mixed signal sequence. All forward and reverse sequences were checked by eye and priming sites were trimmed using Geneious Pro v. 3.6.1 (http://www.geneious.com). Trimmed sequences were compared to BOLD (FISH-BOL) and BLAST (GenBank) databases to identify them to the lowest taxonomic category possible (e.g. genus and/or species). In addition, we developed a character-based identification key (S2 Table) for all CITES-listed species based on the methodology by Fields et al. (2015). We downloaded and trimmed DNA sequences from all CITES-listed species and created a consensus sequence, which then was compared to its closest relatives based on previous phylogenetic studies [20]. Next, we determined all the nucleotides at multiple positions that differentiate each CITES-listed species from their closest relatives, which from now on will be referred to as the compound character attribute (cCA). Tissues were considered identifiable to the species level when the closest match in BLAST showed at least 2 bp differences to our target sequence, when BOLD returned a 100% species level match, and by using the cCA key (S2 Table).

## Results and discussion

Our initial trial of the mini barcode sequences obtained from processed fins (i.e., when both 150R and 474F were obtained) resulted in the positive identification of at least 22 species and three species complexes [20], comprising a wide range of sharks and one batoid (Table 2). These species/complexes were from four orders, eight families, and fifteen genera (Table 2). Based on the wide taxonomic spectrum observed in this trial we suggest that this assay has the potential to amplify both mini-barcodes from processed fins of most, if not all, shark species in trade. When both amplicons are sequenced the assay improves on [10] because both mini-barcodes enable the identification to species of all CITES-listed sharks, including the oceanic whitetip shark (*C*. *longimanus*), that can only be identified to genus with the 150R amplicon, and provides more support (i.e. >2 bps) for the identification of the silky shark (*Carcharhinus falciformis*). Additionally, the Shark474 alone can resolve some of these uncertainties (e.g. *C*. *longimanus* identification), therefore the amplification of the Shark474 (-+- in Table 3) also improves on [10]. Using both the Shark150 and Shark474 mini barcodes together also reduces identification uncertainties in other requiem sharks (*Carcharhinus spp*.; S1 Alignment File) described by [10], enabling more straightforward identification of processed products from one of the most common shark genera in trade (S3 Table). Moreover, fragments might not be fully sequenced, and ambiguities within the sequences are common due to the degraded nature of these products. Therefore, even when differences between closely related species might be 2 bps or more within the Shark150, combining both amplicons allowed for a better identification resolution due to shorter sequences and ambiguous nucleotides. There are a number of taxonomic uncertainties and unresolved species complexes within this group that still need revision before all *Carcharhinus* products in trade can be identified to the species level [20].

**Table 2:** Samples identified from processed fins from the Guangzhou and Honk Kong SAR markets and species where both mini-barcode amplicons amplified successfully in a single reaction.

Order	Family	Species	+/+ (N)
Lamniformes	Alopiidae	Alopias superciliosus**	1
Carcharhiniformes	Carcharhinidae	Blacktip complex*	10
Carcharhiniformes	Carcharhinidae	*Carcharhinus brevipinna*	10
Carcharhiniformes	Carcharhinidae	Carcharhinus falciformis**	10
Carcharhiniformes	Carcharhinidae	*Carcharhinus sorrah*	10
Carcharhiniformes	Carcharhinidae	*Galeocerdo cuvier*	1
Carcharhiniformes	Hemigaleidae	*Hemigaleus australiensis*	1
Carcharhiniformes	Hemigaleidae	*Hemipristis elongata*	1
Lamniformes	Lamnidae	*Isurus oxyrhinchus*	1
Carcharhiniformes	Carcharhinidae	*Prionace glauca*	10
Carcharhiniformes	Carcharhinidae	*Rhizoprionodon acutus*	10
Carcharhiniformes	Carcharhinidae	*Rhizoprionodon porosus/terraenovae*	1
Carcharhiniformes	Sphyrnidae	Sphyrna lewini**	10
Carcharhiniformes	Sphyrnidae	Sphyrna zygaena**	5
Carcharhiniformes	Carcharhinidae	*Triaenodon obesus*	1
Carcharhiniformes	Carcharhinidae	*Carcharhinus amboinensis*	10
Carcharhiniformes	Carcharhinidae	*Carcharhinus galapagensis/obscurus*	10
Carcharhiniformes	Carcharhinidae	*Carcharhinus leucas*	5
Carcharhiniformes	Carcharhinidae	Carcharhinus longimanus**	1
Orectolobiformes	Hemiscylliidae	*Chiloscyllium punctatum*	1
Carcharhiniformes	Carcharhinidae	*Lamiopsis temminckii*	1
Carcharhiniformes	Triakidae	*Mustelus mustelus*	1
Carcharhiniformes	Carcharhinidae	*Negaprion acutidens*	1
Rajiformes	Rhinidae	*Rhynchobatus djiddensis*	1
Carcharhiniformes	Sphyrnidae	Sphyrna mokarran**	1

* Blacktip complex denotes the species complex determined by *Carcharhinus limbatus*, *C*. *amblyrhinchoides*, *C*. *leiodon*, and *C*. *tilstoni*.

** denotes CITES-listed species in Appendix II.

**Table 3:** Five possible amplification options of the multiplex PCR assay showing the different amplicons and percentage of the samples from Guangzhou, China (n = 200) that amplified for each option.

COI	Shark474	Shark150	% of samples
+	+	+	0.5%
-	+	+	37.0%
-	+	-	7.0%
-	-	+	40.5%
-	-	-	15%

+ denotes positive amplification, - indicates no amplification.

We used the assay to identify 200 unknown processed fins from Guangzhou, China, to assess its performance in a typical scenario of how it might be used in practice. The assay successfully identified 170 (85%) of these fins to the genus or species level. Thirty samples (15%) either did not amplify or the resulting sequences were of poor quality, impeding a reliable identification (Table 3). These samples were run multiple times with the same result, likely due to poor DNA quality. Only 0.5% of these fins yielded the full COI sequence, which is consistent with our previous trials with this type of tissue that contains highly degraded DNA (Table 3). More than a third (37%) yielded high quality sequences for both the Shark150 and Shark474 mini-barcodes, comprising nearly half (44.7%) of the fins that were amplified (Table 3). The remaining fins yielded only the 474F (7% of total) or 150R amplicon (40.5% of total, Table 3). The higher frequency of successful amplification and sequencing of the 150R amplicon in this trial most likely results this fragment being more likely to amplify well from degraded tissue when compared to the larger 474F amplicon.

Our assay also amplified a total of thirteen samples from the liver oil pills, shark fin soup and skin care products tested (65%; Table 4). Even though this protocol was able to detect the presence of shark DNA traces in these kinds of product, it does not necessarily mean that further development isn’t required to use them in law enforcement contexts. Individual oil, soup, and cosmetic products could be composed of mixtures of more than one species. We did not test the assay for possible amplification biases, which could lead to identification problems with mixed species products. We suggest using our primers for next-generation sequencing in a metagenomics context in order to determine if more than one species is present in products of this kind.

**Table 4:** Shark species identified in other shark products with potentially degraded DNA.

Sample #	Product type	Species
1	Shark fin soup	*Prionace glauca*
2	Shark fin soup	*Sphyrna sp*.
3	Shark fin soup	No Amplification
4	Shark fin soup	No Amplification
5	Liver oil pill	*Carcharhinus sp*.
6	Liver oil pill	*Carcharhinus sp*.
7	Liver oil pill	Blacktip complex*
8	Liver oil pill	*Prionace glauca*
9	Facial foam	*Prionace glauca*
10	Facial foam	Sphyrna lewini**
11	Facial foam	Blacktip complex*
12	Facial foam	No Amplification
13	Facial mask	*Carcharhinus sp*.
14	Facial mask	*Prionace glauca*
15	Facial mask	No Amplification
16	Facial mask	No Amplification
17	Facial oil	*Rhynchobatus sp*.
18	Facial oil	*Carcharhinus sp*.
19	Facial oil	No Amplification
20	Facial oil	No Amplification

*Blacktip complex denotes the species complex determined by *Carcharhinus limbatus*, *C*. *amblyrhinchoides*, *C*. *leiodon*, and *C*. *tilstoni*.

** denotes CITES-listed species in Appendix II.

Individually, the Shark150 and the Shark474 amplified 78% and 44.5% of the time respectively, while only one sample amplified with the full COI amplicon. Our performance trials indicate that use of this assay frequently yields one or two mini-barcodes for highly processed shark tissues in a variety of traded products. When only one mini-barcode is obtained, it is typically the smaller 150R and can still be used identify many species on its own (e.g., blue shark, *Prionace glauca*, the most common species in the fin trade [4], and ten of the twelve CITES listed sharks [10]) and the remainder to genus. When both mini-barcodes are obtained, which occurred for around half the products that amplified, all CITES listed species and most *Carcharhinus* species can be identified to species. The traceability and regulation of shark products along the supply chain is a crucial step in order to improve the sustainability of shark fisheries and to recover imperiled species [21]. The applications of the multiplex assay described here comprise (i) the monitoring and enforcement of regulations implemented for certain species at all governance levels (e.g. national, regional, international), and (ii) the monitoring of highly processed products commonly found in trade such as fins, fin soup, and liver oil.

## Supporting information

S1 Table Carcharhinus samples used to design Shark474F. (DOCX)

S2 Table Compound character attributes (cCA) for CITES-listed shark species. (XLSX)

S3 Table Number of nucleotide differences between species of the *Carcharhinus* genus in samples from Guangzhou, China, at the Shark150 region (above diagonal) and Shark474 region (below diagonal). Highlighted cells and number in bold depict two or less nucleotide differences.
(XLSX)

S1 Alignment File Alignment of the Shark150 and Shark474 for common Carcharhinus spp. found in Asian shark fin markets. (TXT)
