# Supplementary material for: A multiplex PCR mini-barcode assay to identify processed shark products in the global trade
Source: PLoS One. 2017 Oct 11;12(10):e0185368. doi: 10.1371/journal.pone.0185368 (PMC5636071; doi:10.1371/journal.pone.0185368)
Supplement: S1 Table — (DOCX) [file pone.0185368.s001.docx]

S1 Table. Carcharhinus samples used to design Shark474F.

| **gi number** | **Accession number** | **Species** |  |
| --- | --- | --- | --- |
| gi\|384369567 | gb JQ654712.1 | Carcharhinus altimus |  |
| gi\|166836839 | gb EU398589.1 | Carcharhinus altimus |  |
| gi\|166836837 | gb EU398588.1 | Carcharhinus altimus |  |
| gi\|261874059 | gb FJ519045.1 | Carcharhinus altimus | |
| gi\|261874061 | gb FJ519046.1 | Carcharhinus altimus |  |
| gi\|166836835 | gb EU398587.1 | Carcharhinus altimus |  |
| gi\|261874063 | gb FJ519047.1 | Carcharhinus altimus |  |
| gi\|261874069 | gb FJ519050.1 | Carcharhinus altimus |  |
| gi\|261874065 | gb FJ519048.1 | Carcharhinus altimus |  |
| gi\|261874067 | gb FJ519049.1 | Carcharhinus altimus |  |
| gi\|359817466 | gb JN641207.1 | Carcharhinus altimus |  |
| gi\|359817464 | gb JN641206.1 | Carcharhinus altimus |  |
| gi\|547103946 | gb KF606805.1 | Carcharhinus albimarginatus |  |
| gi\|547104819 | gb KF606843.1 | Carcharhinus albimarginatus |  |
| gi\|547103518 | gb KF606784.1 | Carcharhinus albimarginatus |  |
| gi\|261874357 | gb FJ519194.1 | Carcharhinus albimarginatus |  |
| gi\|261874359 | gb FJ519195.1 | Carcharhinus albimarginatus |  |
| gi\|261874351 | gb FJ519191.1 | Carcharhinus albimarginatus |  |
| gi\|261874353 | gb FJ519192.1 | Carcharhinus albimarginatus |  |
| gi\|166836831 | gb EU398585.1 | Carcharhinus albimarginatus |  |
| gi\|166836829 | gb EU398584.1 | Carcharhinus albimarginatus |  |
| gi\|166836825 | gb EU398582.1 | Carcharhinus albimarginatus |  |
| gi\|261874651 | gb FJ519341.1 | Carcharhinus albimarginatus |  |
| gi\|166836833 | gb EU398586.1 | Carcharhinus albimarginatus |  |
| gi\|166836827 | gb EU398583.1 | Carcharhinus albimarginatus |  |
| gi\|564735827 | gb KF728380.1 | Carcharhinus acronotus |  |
| gi\|554521252 | gb KF461148.1 | Carcharhinus acronotus |  |
| gi\|261874049 | gb FJ519040.1 | Carcharhinus acronotus |  |
| gi\|261874051 | gb FJ519041.1 | Carcharhinus acronotus |  |
| gi\|261874039 | gb FJ519035.1 | Carcharhinus acronotus |  |
| gi\|261874045 | gb FJ519038.1 | Carcharhinus acronotus |  |
| gi\|261874041 | gb FJ519036.1 | Carcharhinus acronotus |  |
| gi\|261874043 | gb FJ519037.1 | Carcharhinus acronotus |  |
| gi\|261874331 | gb FJ519181.1 | Carcharhinus acronotus |  |
| gi\|116607907 | gb DQ884978.1 | Carcharhinus amboinensis |  |
| gi\|116607755 | gb DQ885075.1 | Carcharhinus amboinensis |  |
| gi\|556910896 | gb KF590340.1 | Carcharhinus amboinensis |  |
| gi\|116607759 | gb DQ885077.1 | Carcharhinus amboinensis |  |
| gi\|116607757 | gb DQ885076.1 | Carcharhinus amboinensis |  |
| gi\|328484540 | gb JF493045.1 | Carcharhinus amboinensis |  |
| gi\|166836861 | gb EU398600.1 | Carcharhinus amboinensis |  |
| gi\|261874077 | gb FJ519054.1 | Carcharhinus amboinensis |  |
| gi\|556910998 | gb KF590391.1 | Carcharhinus amblyrhynchos |  |
| gi\|556910990 | gb KF590387.1 | Carcharhinus amblyrhynchos |  |
| gi\|556910986 | gb KF590385.1 | Carcharhinus amblyrhynchos |  |
| gi\|166836851 | gb EU398595.1 | Carcharhinus amblyrhynchos |  |
| gi\|166836849 | gb EU398594.1 | Carcharhinus amblyrhynchos |  |
| gi\|166836857 | gb EU398598.1 | Carcharhinus amblyrhynchos |  |
| gi\|166836855 | gb EU398597.1 | Carcharhinus amblyrhynchos |  |
| gi\|166836853 | gb EU398596.1 | Carcharhinus amblyrhynchos |  |
| gi\|148373823 | gb EF609308.1 | Carcharhinus amblyrhynchos |  |
| gi\|261874677 | gb FJ519354.1 | Carcharhinus amblyrhynchos |  |
| gi\|261874685 | gb FJ519358.1 | Carcharhinus amblyrhynchos |  |
| gi\|261874665 | gb FJ519348.1 | Carcharhinus amblyrhynchos |  |
| gi\|261874671 | gb FJ519351.1 | Carcharhinus amblyrhynchos |  |
| gi\|261874363 | gb FJ519197.1 | Carcharhinus amblyrhynchos |  |
| gi\|261874361 | gb FJ519196.1 | Carcharhinus amblyrhynchos |  |
| gi\|261874663 | gb FJ519347.1 | Carcharhinus amblyrhynchos |  |
| gi\|261874369 | gb FJ519200.1 | Carcharhinus amblyrhynchos |  |
| gi\|261874371 | gb FJ519201.1 | Carcharhinus amblyrhynchos |  |
| gi\|261874367 | gb FJ519199.1 | Carcharhinus amblyrhynchos |  |
| gi\|593023763 | gb KF956523.1 | Carcharhinus amblyrhynchoides |  |
| gi\|291165449 | gb GQ227287.1 | Carcharhinus amblyrhynchoides |  |
| gi\|148373821 | gb EF609307.1 | Carcharhinus amblyrhynchoides |  |
| gi\|336327325 | gb JN082185.1 | Carcharhinus amblyrhynchoides |  |
| gi\|336280766 | gb JN034898.1 | Carcharhinus amblyrhynchoides |  |
| gi\|336280764 | gb JN034897.1 | Carcharhinus amblyrhynchoides |  |
| gi\|336280762 | gb JN034896.1 | Carcharhinus amblyrhynchoides |  |
| gi\|336327323 | gb JN082184.1 | Carcharhinus amblyrhynchoides |  |
| gi\|336327317 | gb JN082181.1 | Carcharhinus amblyrhynchoides |  |
| gi\|336327319 | gb JN082182.1 | Carcharhinus amblyrhynchoides |  |
| gi\|336327321 | gb JN082183.1 | Carcharhinus amblyrhynchoides |  |
| gi\|148373825 | gb EF609309.1 | Carcharhinus cautus |  |
| gi\|261874111 | gb FJ519071.1 | Carcharhinus cautus |  |
| gi\|166836869 | gb EU398604.1 | Carcharhinus cautus |  |
| gi\|166836875 | gb EU398607.1 | Carcharhinus cautus |  |
| gi\|166836873 | gb EU398606.1 | Carcharhinus cautus |  |
| gi\|426202505 | gb KC175450.1 | Carcharhinus brevipinna |  |
| gi\|556911026 | gb KF590405.1 | Carcharhinus brevipinna |  |
| gi\|556910996 | gb KF590390.1 | Carcharhinus brevipinna |  |
| gi\|556910900 | gb KF590342.1 | Carcharhinus brevipinna |  |
| gi\|554521254 | gb KF461149.1 | Carcharhinus brevipinna |  |
| gi\|261875181 | gb FJ519606.1 | Carcharhinus brevipinna |  |
| gi\|261874109 | gb FJ519070.1 | Carcharhinus brevipinna |  |
| gi\|575933732 | gb KF793760.1 | Carcharhinus brevipinna |  |
| gi\|166836867 | gb EU398603.1 | Carcharhinus brevipinna |  |
| gi\|166836865 | gb EU398602.1 | Carcharhinus brevipinna |  |
| gi\|166836863 | gb EU398601.1 | Carcharhinus brevipinna |  |
| gi\|261874093 | gb FJ519062.1 | Carcharhinus brevipinna |  |
| gi\|261874095 | gb FJ519063.1 | Carcharhinus brevipinna |  |
| gi\|556910802 | gb KF590293.1 | Carcharhinus brevipinna |  |
| gi\|328484568 | gb JF493059.1 | Carcharhinus brevipinna |  |
| gi\|306993253 | gb HQ171636.1 | Carcharhinus brevipinna |  |
| gi\|306993213 | gb HQ171616.1 | Carcharhinus brevipinna |  |
| gi\|261874103 | gb FJ519067.1 | Carcharhinus brevipinna |  |
| gi\|328484560 | gb JF493055.1 | Carcharhinus brevipinna |  |
| gi\|306993247 | gb HQ171633.1 | Carcharhinus brevipinna |  |
| gi\|296837028 | gb GU804990.1 | Carcharhinus brevipinna |  |
| gi\|306993249 | gb HQ171634.1 | Carcharhinus brevipinna |  |
| gi\|306993257 | gb HQ171638.1 | Carcharhinus brevipinna |  |
| gi\|306993263 | gb HQ171641.1 | Carcharhinus brevipinna |  |
| gi\|556910988 | gb KF590386.1 | Carcharhinus brevipinna |  |
| gi\|547104652 | gb KF606833.1 | Carcharhinus brevipinna |  |
| gi\|261874097 | gb FJ519064.1 | Carcharhinus brevipinna |  |
| gi\|306993227 | gb HQ171623.1 | Carcharhinus brevipinna |  |
| gi\|306993251 | gb HQ171635.1 | Carcharhinus brevipinna |  |
| gi\|575933702 | gb KF793745.1 | Carcharhinus brevipinna |  |
| gi\|328484554 | gb JF493052.1 | Carcharhinus brachyurus |  |
| gi\|328484548 | gb JF493049.1 | Carcharhinus brachyurus |  |
| gi\|328484550 | gb JF493050.1 | Carcharhinus brachyurus |  |
| gi\|261874083 | gb FJ519057.1 | Carcharhinus brachyurus |  |
| gi\|261874087 | gb FJ519059.1 | Carcharhinus brachyurus |  |
| gi\|261874085 | gb FJ519058.1 | Carcharhinus brachyurus |  |
| gi\|328484552 | gb JF493051.1 | Carcharhinus brachyurus |  |
| gi\|547104920 | gb KF606849.1 | Carcharhinus brachyurus |  |
| gi\|556911254 | gb KF590519.1 | Carcharhinus coatesi |  |
| gi\|291165451 | gb GQ227288.1 | Carcharhinus dussumieri |  |
| gi\|166836881 | gb EU398610.1 | Carcharhinus dussumieri |  |
| gi\|166836877 | gb EU398608.1 | Carcharhinus dussumieri |  |
| gi\|166836879 | gb EU398609.1 | Carcharhinus dussumieri |  |
| gi\|70724096 | gb DQ108301.1 | Carcharhinus dussumieri |  |
| gi\|70724098 | gb DQ108302.1 | Carcharhinus dussumieri |  |
| gi\|261874121 | gb FJ519076.1 | Carcharhinus dussumieri |  |
| gi\|261874113 | gb FJ519072.1 | Carcharhinus dussumieri |  |
| gi\|261874115 | gb FJ519073.1 | Carcharhinus dussumieri |  |
| gi\|526131403 | gb KC840953.1 | Carcharhinus falciformis |  |
| gi\|261874127 | gb FJ519079.1 | Carcharhinus falciformis |  |
| gi\|261874129 | gb FJ519080.1 | Carcharhinus falciformis |  |
| gi\|556911248 | gb KF590516.1 | Carcharhinus falciformis |  |
| gi\|556911246 | gb KF590515.1 | Carcharhinus falciformis |  |
| gi\|556911226 | gb KF590505.1 | Carcharhinus falciformis |  |
| gi\|556911212 | gb KF590498.1 | Carcharhinus falciformis |  |
| gi\|556911204 | gb KF590494.1 | Carcharhinus falciformis |  |
| gi\|556911186 | gb KF590485.1 | Carcharhinus falciformis |  |
| gi\|556911128 | gb KF590456.1 | Carcharhinus falciformis |  |
| gi\|556911074 | gb KF590429.1 | Carcharhinus falciformis |  |
| gi\|556911072 | gb KF590428.1 | Carcharhinus falciformis |  |
| gi\|556911068 | gb KF590426.1 | Carcharhinus falciformis |  |
| gi\|556911064 | gb KF590424.1 | Carcharhinus falciformis |  |
| gi\|556911062 | gb KF590423.1 | Carcharhinus falciformis |  |
| gi\|556911038 | gb KF590411.1 | Carcharhinus falciformis |  |
| gi\|556911000 | gb KF590392.1 | Carcharhinus falciformis |  |
| gi\|556910962 | gb KF590373.1 | Carcharhinus falciformis |  |
| gi\|556910950 | gb KF590367.1 | Carcharhinus falciformis |  |
| gi\|556910946 | gb KF590365.1 | Carcharhinus falciformis |  |
| gi\|556910942 | gb KF590363.1 | Carcharhinus falciformis |  |
| gi\|556910940 | gb KF590362.1 | Carcharhinus falciformis |  |
| gi\|556910906 | gb KF590345.1 | Carcharhinus falciformis |  |
| gi\|556910904 | gb KF590344.1 | Carcharhinus falciformis |  |
| gi\|556910868 | gb KF590326.1 | Carcharhinus falciformis |  |
| gi\|556910866 | gb KF590325.1 | Carcharhinus falciformis |  |
| gi\|556910864 | gb KF590324.1 | Carcharhinus falciformis |  |
| gi\|556910824 | gb KF590304.1 | Carcharhinus falciformis |  |
| gi\|556910822 | gb KF590303.1 | Carcharhinus falciformis |  |
| gi\|556910818 | gb KF590301.1 | Carcharhinus falciformis |  |
| gi\|556910816 | gb KF590300.1 | Carcharhinus falciformis |  |
| gi\|556910812 | gb KF590298.1 | Carcharhinus falciformis |  |
| gi\|556910810 | gb KF590297.1 | Carcharhinus falciformis |  |
| gi\|556910808 | gb KF590296.1 | Carcharhinus falciformis |  |
| gi\|556910806 | gb KF590295.1 | Carcharhinus falciformis |  |
| gi\|556910804 | gb KF590294.1 | Carcharhinus falciformis |  |
| gi\|556910800 | gb KF590292.1 | Carcharhinus falciformis |  |
| gi\|526131401 | gb KC840952.1 | Carcharhinus falciformis |  |
| gi\|166836887 | gb EU398613.1 | Carcharhinus falciformis |  |
| gi\|166836883 | gb EU398611.1 | Carcharhinus falciformis |  |
| gi\|261874135 | gb FJ519083.1 | Carcharhinus falciformis |  |
| gi\|261874137 | gb FJ519084.1 | Carcharhinus falciformis |  |
| gi\|261874131 | gb FJ519081.1 | Carcharhinus falciformis |  |
| gi\|556911210 | gb KF590497.1 | Carcharhinus falciformis |  |
| gi\|556911202 | gb KF590493.1 | Carcharhinus falciformis |  |
| gi\|556911200 | gb KF590492.1 | Carcharhinus falciformis |  |
| gi\|556911198 | gb KF590491.1 | Carcharhinus falciformis |  |
| gi\|556911188 | gb KF590486.1 | Carcharhinus falciformis |  |
| gi\|556911184 | gb KF590484.1 | Carcharhinus falciformis |  |
| gi\|556910958 | gb KF590371.1 | Carcharhinus falciformis |  |
| gi\|556910956 | gb KF590370.1 | Carcharhinus falciformis |  |
| gi\|556910954 | gb KF590369.1 | Carcharhinus falciformis |  |
| gi\|556910952 | gb KF590368.1 | Carcharhinus falciformis |  |
| gi\|556910944 | gb KF590364.1 | Carcharhinus falciformis |  |
| gi\|261874133 | gb FJ519082.1 | Carcharhinus falciformis |  |
| gi\|556910960 | gb KF590372.1 | Carcharhinus falciformis |  |
| gi\|576864484 | gb KF801102.1 | Carcharhinus falciformis |  |
| gi\|556911238 | gb KF590511.1 | Carcharhinus falciformis |  |
| gi\|556910948 | gb KF590366.1 | Carcharhinus falciformis |  |
| gi\|556911066 | gb KF590425.1 | Carcharhinus falciformis |  |
| gi\|166836885 | gb EU398612.1 | Carcharhinus falciformis |  |
| gi\|237847778 | gb FJ895094.1 | Carcharhinus falciformis |  |
| gi\|556910782 | gb KF590283.1 | Carcharhinus falciformis |  |
| gi\|547104256 | gb KF606816.1 | Carcharhinus falciformis |  |
| gi\|547103772 | gb KF606795.1 | Carcharhinus falciformis |  |
| gi\|547103866 | gb KF606802.1 | Carcharhinus falciformis |  |
| gi\|547105109 | gb KF606860.1 | Carcharhinus falciformis |  |
| gi\|547103918 | gb KF606804.1 | Carcharhinus falciformis |  |
| gi\|547103657 | gb KF606790.1 | Carcharhinus falciformis |  |
| gi\|547103160 | gb KF606770.1 | Carcharhinus falciformis |  |
| gi\|166836891 | gb EU398615.1 | Carcharhinus fitzroyensis |  |
| gi\|148373827 | gb EF609310.1 | Carcharhinus fitzroyensis |  |
| gi\|384369571 | gb JQ654714.1 | Carcharhinus galapagensis |  |
| gi\|261873793 | gb FJ518912.1 | Carcharhinus galapagensis |  |
| gi\|261873795 | gb FJ518913.1 | Carcharhinus galapagensis |  |
| gi\|261874147 | gb FJ519089.1 | Carcharhinus galapagensis |  |
| gi\|261873789 | gb FJ518910.1 | Carcharhinus galapagensis |  |
| gi\|556911048 | gb KF590416.1 | Carcharhinus galapagensis |  |
| gi\|556911046 | gb KF590415.1 | Carcharhinus galapagensis |  |
| gi\|261874159 | gb FJ519095.1 | Carcharhinus galapagensis |  |
| gi\|261873803 | gb FJ518917.1 | Carcharhinus galapagensis |  |
| gi\|547103398 | gb KF606780.1 | Carcharhinus galapagensis |  |
| gi\|547103049 | gb KF606767.1 | Carcharhinus galapagensis |  |
| gi\|547103227 | gb KF606772.1 | Carcharhinus galapagensis |  |
| gi\|554521260 | gb KF461152.1 | Carcharhinus limbatus |  |
| gi\|261875195 | gb FJ519613.1 | Carcharhinus limbatus |  |
| gi\|261875197 | gb FJ519614.1 | Carcharhinus limbatus |  |
| gi\|383388134 | gb JQ365263.1 | Carcharhinus limbatus |  |
| gi\|383388132 | gb JQ365262.1 | Carcharhinus limbatus |  |
| gi\|383388130 | gb JQ365261.1 | Carcharhinus limbatus |  |
| gi\|383388126 | gb JQ365259.1 | Carcharhinus limbatus |  |
| gi\|556910798 | gb KF590291.1 | Carcharhinus limbatus |  |
| gi\|291165435 | gb GQ227280.1 | Carcharhinus limbatus |  |
| gi\|116607911 | gb DQ884980.1 | Carcharhinus limbatus |  |
| gi\|116607763 | gb DQ885079.1 | Carcharhinus limbatus |  |
| gi\|116607761 | gb DQ885078.1 | Carcharhinus limbatus |  |
| gi\|556911228 | gb KF590506.1 | Carcharhinus limbatus |  |
| gi\|556911216 | gb KF590500.1 | Carcharhinus limbatus |  |
| gi\|556910980 | gb KF590382.1 | Carcharhinus limbatus |  |
| gi\|556910742 | gb KF590263.1 | Carcharhinus limbatus |  |
| gi\|556910740 | gb KF590262.1 | Carcharhinus limbatus |  |
| gi\|556910738 | gb KF590261.1 | Carcharhinus limbatus |  |
| gi\|556910736 | gb KF590260.1 | Carcharhinus limbatus |  |
| gi\|556910734 | gb KF590259.1 | Carcharhinus limbatus |  |
| gi\|556910730 | gb KF590257.1 | Carcharhinus limbatus |  |
| gi\|556910718 | gb KF590251.1 | Carcharhinus limbatus |  |
| gi\|291165439 | gb GQ227282.1 | Carcharhinus limbatus |  |
| gi\|166836903 | gb EU398621.1 | Carcharhinus limbatus |  |
| gi\|166836905 | gb EU398622.1 | Carcharhinus limbatus |  |
| gi\|261874199 | gb FJ519115.1 | Carcharhinus limbatus |  |
| gi\|261874201 | gb FJ519116.1 | Carcharhinus limbatus |  |
| gi\|116607909 | gb DQ884979.1 | Carcharhinus limbatus |  |
| gi\|359386157 | gb JN989310.1 | Carcharhinus limbatus |  |
| gi\|261874195 | gb FJ519113.1 | Carcharhinus limbatus |  |
| gi\|261874197 | gb FJ519114.1 | Carcharhinus limbatus |  |
| gi\|291165437 | gb GQ227281.1 | Carcharhinus limbatus |  |
| gi\|336327331 | gb JN082188.1 | Carcharhinus limbatus |  |
| gi\|383388128 | gb JQ365260.1 | Carcharhinus limbatus |  |
| gi\|261874189 | gb FJ519110.1 | Carcharhinus limbatus |  |
| gi\|261874191 | gb FJ519111.1 | Carcharhinus limbatus |  |
| gi\|261874193 | gb FJ519112.1 | Carcharhinus limbatus |  |
| gi\|194738571 | gb EU541307.1 | Carcharhinus limbatus |  |
| gi\|303305608 | gb HM231107.1 | Carcharhinus limbatus |  |
| gi\|336327329 | gb JN082187.1 | Carcharhinus limbatus |  |
| gi\|547103422 | gb KF606781.1 | Carcharhinus limbatus |  |
| gi\|547104448 | gb KF606822.1 | Carcharhinus limbatus |  |
| gi\|384369563 | gb JQ654710.1 | Carcharhinus limbatus |  |
| gi\|328484576 | gb JF493063.1 | Carcharhinus leucas |  |
| gi\|328484574 | gb JF493062.1 | Carcharhinus leucas |  |
| gi\|328484572 | gb JF493061.1 | Carcharhinus leucas |  |
| gi\|328484570 | gb JF493060.1 | Carcharhinus leucas |  |
| gi\|261873971 | gb FJ519001.1 | Carcharhinus leucas |  |
| gi\|261873973 | gb FJ519002.1 | Carcharhinus leucas |  |
| gi\|211909095 | gb EU818710.1 | Carcharhinus leucas |  |
| gi\|148373829 | gb EF609311.1 | Carcharhinus leucas |  |
| gi\|261873981 | gb FJ519006.1 | Carcharhinus leucas |  |
| gi\|554521258 | gb KF461151.1 | Carcharhinus leucas |  |
| gi\|261873967 | gb FJ518999.1 | Carcharhinus leucas |  |
| gi\|261873969 | gb FJ519000.1 | Carcharhinus leucas |  |
| gi\|556911220 | gb KF590502.1 | Carcharhinus leucas |  |
| gi\|261873983 | gb FJ519007.1 | Carcharhinus leucas |  |
| gi\|547104945 | gb KF606851.1 | Carcharhinus leucas |  |
| gi\|557637653 | gb KF646785.1 | Carcharhinus leucas |  |
| gi\|336280776 | gb JN034903.1 | Carcharhinus leiodon |  |
| gi\|336280774 | gb JN034902.1 | Carcharhinus leiodon |  |
| gi\|336280772 | gb JN034901.1 | Carcharhinus leiodon |  |
| gi\|336280770 | gb JN034900.1 | Carcharhinus leiodon |  |
| gi\|336280768 | gb JN034899.1 | Carcharhinus leiodon |  |
| gi\|556911224 | gb KF590504.1 | Carcharhinus longimanus |  |
| gi\|556911070 | gb KF590427.1 | Carcharhinus longimanus |  |
| gi\|556911042 | gb KF590413.1 | Carcharhinus longimanus |  |
| gi\|556911040 | gb KF590412.1 | Carcharhinus longimanus |  |
| gi\|556910708 | gb KF590246.1 | Carcharhinus longimanus |  |
| gi\|556910700 | gb KF590242.1 | Carcharhinus longimanus |  |
| gi\|166836913 | gb EU398626.1 | Carcharhinus longimanus |  |
| gi\|556911218 | gb KF590501.1 | Carcharhinus longimanus |  |
| gi\|294988842 | gb GU440259.1 | Carcharhinus longimanus |  |
| gi\|261873809 | gb FJ518920.1 | Carcharhinus longimanus |  |
| gi\|261873811 | gb FJ518921.1 | Carcharhinus longimanus |  |
| gi\|261873807 | gb FJ518919.1 | Carcharhinus longimanus |  |
| gi\|261873825 | gb FJ518928.1 | Carcharhinus longimanus |  |
| gi\|261875207 | gb FJ519619.1 | Carcharhinus longimanus |  |
| gi\|261874205 | gb FJ519118.1 | Carcharhinus longimanus |  |
| gi\|547104004 | gb KF606806.1 | Carcharhinus longimanus |  |
| gi\|547104165 | gb KF606812.1 | Carcharhinus longimanus |  |
| gi\|384369561 | gb JQ654709.1 | Carcharhinus longimanus |  |
| gi\|575933756 | gb KF793772.1 | Carcharhinus melanopterus |  |
| gi\|556910994 | gb KF590389.1 | Carcharhinus melanopterus |  |
| gi\|556910976 | gb KF590380.1 | Carcharhinus melanopterus |  |
| gi\|556910974 | gb KF590379.1 | Carcharhinus melanopterus |  |
| gi\|575933750 | gb KF793769.1 | Carcharhinus melanopterus |  |
| gi\|556910982 | gb KF590383.1 | Carcharhinus melanopterus |  |
| gi\|148373833 | gb EF609313.1 | Carcharhinus melanopterus |  |
| gi\|336327335 | gb JN082190.1 | Carcharhinus melanopterus |  |
| gi\|166836927 | gb EU398633.1 | Carcharhinus melanopterus |  |
| gi\|336327333 | gb JN082189.1 | Carcharhinus melanopterus |  |
| gi\|261874217 | gb FJ519124.1 | Carcharhinus melanopterus |  |
| gi\|261874219 | gb FJ519125.1 | Carcharhinus melanopterus |  |
| gi\|166836921 | gb EU398630.1 | Carcharhinus melanopterus |  |
| gi\|381278762 | gb JQ431553.1 | Carcharhinus melanopterus |  |
| gi\|261874211 | gb FJ519121.1 | Carcharhinus melanopterus |  |
| gi\|261874213 | gb FJ519122.1 | Carcharhinus melanopterus |  |
| gi\|261874209 | gb FJ519120.1 | Carcharhinus melanopterus |  |
| gi\|642988111 | gb KJ720818.1 | Carcharhinus melanopterus |  |
| gi\|359386159 | gb JN989311.1 | Carcharhinus macloti |  |
| gi\|576939671 | gb KF913240.1 | Carcharhinus macloti |  |
| gi\|166836917 | gb EU398628.1 | Carcharhinus macloti |  |
| gi\|148373831 | gb EF609312.1 | Carcharhinus macloti |  |
| gi\|261875085 | gb FJ519558.1 | Carcharhinus plumbeus |  |
| gi\|306993279 | gb HQ171649.1 | Carcharhinus plumbeus |  |
| gi\|261875215 | gb FJ519623.1 | Carcharhinus plumbeus |  |
| gi\|261874279 | gb FJ519155.1 | Carcharhinus plumbeus |  |
| gi\|261874281 | gb FJ519156.1 | Carcharhinus plumbeus |  |
| gi\|328484588 | gb JF493069.1 | Carcharhinus plumbeus |  |
| gi\|328484584 | gb JF493067.1 | Carcharhinus plumbeus |  |
| gi\|261874273 | gb FJ519152.1 | Carcharhinus plumbeus |  |
| gi\|261874265 | gb FJ519148.1 | Carcharhinus plumbeus |  |
| gi\|261874267 | gb FJ519149.1 | Carcharhinus plumbeus |  |
| gi\|261874263 | gb FJ519147.1 | Carcharhinus plumbeus |  |
| gi\|166836939 | gb EU398639.1 | Carcharhinus plumbeus |  |
| gi\|166836937 | gb EU398638.1 | Carcharhinus plumbeus |  |
| gi\|556910670 | gb KF590227.1 | Carcharhinus plumbeus |  |
| gi\|556910666 | gb KF590225.1 | Carcharhinus plumbeus |  |
| gi\|556910660 | gb KF590222.1 | Carcharhinus plumbeus |  |
| gi\|306993283 | gb HQ171651.1 | Carcharhinus plumbeus |  |
| gi\|384369565 | gb JQ654711.1 | Carcharhinus plumbeus |  |
| gi\|547103721 | gb KF606793.1 | Carcharhinus plumbeus |  |
| gi\|662020340 | gb KJ740750.1 | Carcharhinus plumbeus |  |
| gi\|261874255 | gb FJ519143.1 | Carcharhinus perezii |  |
| gi\|261874243 | gb FJ519137.1 | Carcharhinus perezii |  |
| gi\|261874245 | gb FJ519138.1 | Carcharhinus perezii |  |
| gi\|556911262 | gb KF590523.1 | Carcharhinus obscurus |  |
| gi\|70724076 | gb DQ108291.1 | Carcharhinus obscurus |  |
| gi\|70724106 | gb DQ108306.1 | Carcharhinus obscurus |  |
| gi\|166836929 | gb EU398634.1 | Carcharhinus obscurus |  |
| gi\|116607917 | gb DQ884983.1 | Carcharhinus obscurus |  |
| gi\|328484582 | gb JF493066.1 | Carcharhinus obscurus |  |
| gi\|261874231 | gb FJ519131.1 | Carcharhinus obscurus |  |
| gi\|261873827 | gb FJ518929.1 | Carcharhinus obscurus |  |
| gi\|261874239 | gb FJ519135.1 | Carcharhinus obscurus |  |
| gi\|306993277 | gb HQ171648.1 | Carcharhinus obscurus |  |
| gi\|116607915 | gb DQ884982.1 | Carcharhinus obscurus |  |
| gi\|456368036 | gb KC470543.1 | Carcharhinus obscurus |  |
| gi\|261874291 | gb FJ519161.1 | Carcharhinus signatus |  |
| gi\|261874285 | gb FJ519158.1 | Carcharhinus signatus |  |
| gi\|261874289 | gb FJ519160.1 | Carcharhinus signatus |  |
| gi\|261874287 | gb FJ519159.1 | Carcharhinus signatus |  |
| gi\|261874293 | gb FJ519162.1 | Carcharhinus signatus |  |
| gi\|261874295 | gb FJ519163.1 | Carcharhinus signatus |  |
| gi\|556911006 | gb KF590395.1 | Carcharhinus sealei |  |
| gi\|556911002 | gb KF590393.1 | Carcharhinus sealei |  |
| gi\|556910968 | gb KF590376.1 | Carcharhinus sealei |  |
| gi\|556910966 | gb KF590375.1 | Carcharhinus sealei |  |
| gi\|556910970 | gb KF590377.1 | Carcharhinus sealei |  |
| gi\|166836941 | gb EU398640.1 | Carcharhinus sealei |  |
| gi\|166836943 | gb EU398641.1 | Carcharhinus sealei |  |
| gi\|556910972 | gb KF590378.1 | Carcharhinus sealei |  |
| gi\|166836945 | gb EU398642.1 | Carcharhinus sealei |  |
| gi\|261874283 | gb FJ519157.1 | Carcharhinus porosus |  |
| gi\|291165443 | gb GQ227284.1 | Carcharhinus tilstoni |  |
| gi\|261874195 | gb FJ519113.1 | Carcharhinus limbatus |  |
| gi\|261874197 | gb FJ519114.1 | Carcharhinus limbatus |  |
| gi\|70724060 | gb DQ108283.1 | Carcharhinus tilstoni |  |
| gi\|70724086 | gb DQ108296.1 | Carcharhinus tilstoni |  |
| gi\|291165447 | gb GQ227286.1 | Carcharhinus tilstoni |  |
| gi\|291165445 | gb GQ227285.1 | Carcharhinus tilstoni |  |
| gi\|291165441 | gb GQ227283.1 | Carcharhinus tilstoni |  |
| gi\|261874329 | gb FJ519180.1 | Carcharhinus tilstoni |  |
| gi\|70724090 | gb DQ108298.1 | Carcharhinus tilstoni |  |
| gi\|261874327 | gb FJ519179.1 | Carcharhinus tilstoni |  |
| gi\|70724088 | gb DQ108297.1 | Carcharhinus tilstoni |  |
| gi\|261874201 | gb FJ519116.1 | Carcharhinus limbatus |  |
| gi\|261874319 | gb FJ519175.1 | Carcharhinus tilstoni |  |
| gi\|261874321 | gb FJ519176.1 | Carcharhinus tilstoni |  |
| gi\|261874315 | gb FJ519173.1 | Carcharhinus tilstoni |  |
| gi\|526131399 | gb KC840951.1 | Carcharhinus sorrah |  |
| gi\|70724078 | gb DQ108292.1 | Carcharhinus sorrah |  |
| gi\|70724082 | gb DQ108294.1 | Carcharhinus sorrah |  |
| gi\|261874311 | gb FJ519171.1 | Carcharhinus sorrah |  |
| gi\|261874313 | gb FJ519172.1 | Carcharhinus sorrah |  |
| gi\|70724080 | gb DQ108293.1 | Carcharhinus sorrah |  |
| gi\|556911132 | gb KF590458.1 | Carcharhinus sorrah |  |
| gi\|556911096 | gb KF590440.1 | Carcharhinus sorrah |  |
| gi\|306993289 | gb HQ171654.1 | Carcharhinus sorrah |  |
| gi\|306993287 | gb HQ171653.1 | Carcharhinus sorrah |  |
| gi\|575933740 | gb KF793764.1 | Carcharhinus sorrah |  |
| gi\|556911130 | gb KF590457.1 | Carcharhinus sorrah |  |
| gi\|526131397 | gb KC840950.1 | Carcharhinus sorrah |  |
| gi\|336280778 | gb JN034904.1 | Carcharhinus sorrah |  |
| gi\|261874309 | gb FJ519170.1 | Carcharhinus sorrah |  |
| gi\|556911104 | gb KF590444.1 | Carcharhinus sorrah |  |
| gi\|556911102 | gb KF590443.1 | Carcharhinus sorrah |  |
| gi\|556911100 | gb KF590442.1 | Carcharhinus sorrah |  |
| gi\|556910984 | gb KF590384.1 | Carcharhinus sorrah |  |
| gi\|556910928 | gb KF590356.1 | Carcharhinus sorrah |  |
| gi\|556910902 | gb KF590343.1 | Carcharhinus sorrah |  |
| gi\|556910754 | gb KF590269.1 | Carcharhinus sorrah |  |
| gi\|556910752 | gb KF590268.1 | Carcharhinus sorrah |  |
| gi\|526131395 | gb KC840949.1 | Carcharhinus sorrah |  |
| gi\|261874303 | gb FJ519167.1 | Carcharhinus sorrah |  |
| gi\|261874305 | gb FJ519168.1 | Carcharhinus sorrah |  |
| gi\|261874307 | gb FJ519169.1 | Carcharhinus sorrah |  |
| gi\|306993303 | gb HQ171661.1 | Carcharhinus sorrah |  |
| gi\|451357407 | gb JX978335.1 | Carcharhinus sorrah |  |
| gi\|557469652 | gb KF612341.1 | Carcharhinus sorrah |  |
